# Arbuscular Mycorrhizal Symbiosis Leads to Differential Regulation of Genes and miRNAs Associated with the Cell Wall in Tomato Leaves

**DOI:** 10.3390/biology11060854

**Published:** 2022-06-02

**Authors:** Ana Belén Mendoza-Soto, Amada Zulé Rodríguez-Corral, Adriana Bojórquez-López, Maylin Cervantes-Rojo, Claudia Castro-Martínez, Melina Lopez-Meyer

**Affiliations:** Departamento Biotecnología Agrícola, Instituto Politécnico Nacional, CIIDIR-Sinaloa, Blv. Juan de Dios Bátiz 250, Guasave 81000, Mexico; mendozasotoab@gmail.com (A.B.M.-S.); amada1551@gmail.com (A.Z.R.-C.); adriana.26.aybl@gmail.com (A.B.-L.); maylin.rojo.kc@hotmail.com (M.C.-R.); clcastro@ipn.mx (C.C.-M.)

**Keywords:** arbuscular mycorrhiza, priming, tomato, defense, symbiosis, miRNA, cell wall

## Abstract

**Simple Summary:**

Tomato can interact with arbuscular mycorrhizal fungi (AMF) to form a symbiotic association called arbuscular mycorrhiza. This symbiosis, in addition to providing nutritional benefits to plants, induces a plant defense response against biotic and abiotic stresses locally in the roots, and systemically throughout the entire plant. However, the mechanisms underlying these conferred systemic resistance-induced responses are largely unknown. This work aimed to identify which regulatory molecules could be involved in the response mechanisms elicited during priming. The findings presented here provide valuable information on the molecules that could participate in these responses, with the aim of elucidating the whole mechanism.

**Abstract:**

Arbuscular mycorrhizal symbiosis is an association that provides nutritional benefits to plants. Importantly, it induces a physiological state allowing plants to respond to a subsequent pathogen attack in a more rapid and intense manner. Consequently, mycorrhiza-colonized plants become less susceptible to root and shoot pathogens. This study aimed to identify some of the molecular players and potential mechanisms related to the onset of defense priming by mycorrhiza colonization, as well as miRNAs that may act as regulators of priming genes. The upregulation of cellulose synthases, pectinesterase inhibitors, and xyloglucan endotransglucosylase/hydrolase, as well as the downregulation of a pectinesterase, suggest that the modification and reinforcement of the cell wall may prime the leaves of mycorrhizal plants to react faster and stronger to subsequent pathogen attack. This was confirmed by the findings of miR164a-3p, miR164a-5p, miR171e-5p, and miR397, which target genes and are also related to the biosynthesis or modification of cell wall components. Our findings support the hypothesis that the reinforcement or remodeling of the cell wall and cuticle could participate in the priming mechanism triggered by mycorrhiza colonization, by strengthening the first physical barriers upstream of the pathogen encounter.

## 1. Introduction

Tomato plants can establish symbiotic interactions with arbuscular mycorrhizal fungi (AMF) to give rise to a mutualistic association called arbuscular mycorrhiza, which can occur between the majority of land plants and fungi within the phylum Glomeromycota [1]. This interaction takes place in the roots, where the fungus colonizes the cortex and obtains carbon compounds in the form of carbohydrates and lipids from the plant. These components are required to complete the fungus life cycle while facilitating the transfer of mineral nutrients, such as phosphate, to the root cells through arbuscules, which are differentiated and highly branched intracellular fungal structures [2]. In addition to the nutritional benefit, it has been observed that mycorrhiza colonization induces tolerance to abiotic stresses such as drought, salinity, extreme temperature, herbivory, and metals, as well as biotic stress, such as pathogen attack in roots and aerial organs [3,4,5,6,7,8,9,10,11,12,13,14,15].

It has been postulated that a priming mechanism is activated when plants establish mycorrhizal associations [16]. The increase in basal defenses in colonized plants has been defined as mycorrhiza-induced resistance (MIR), and several studies suggest that this priming mechanism is important for MIR [12,17,18]. The priming mechanism is a physiological state in which the plant is capable of reacting faster and more intensely to the attack of a pathogen [19,20]. This implies that several molecular and biochemical events must occur when the plant establishes this symbiosis, but before the potential attack of a pathogen [12].

The response of mycorrhiza-colonized plants to biotic stress is both local (at the roots) and systemic (throughout the entire plant) [21]. MIR has been documented in a variety of crop species. For example, in tomatoes, Cordier et al. (1998) showed that *Glomus mosseae* can confer protection against *Phytophthora parasitica* in roots [22]. Moreover, in tomato plants colonized with the AMFs *Rhizophagus irregularis* and *Funneliformis* sp., damage caused by the nematode *Nacobbus aberrans* was significantly reduced [23]. In shoots, the symbiosis of tomato with *G. mosseae* and *G. intraradices* provides a systemic and local defense to tomato plants against parasitic *Phytophthora* [24]. Furthermore, (arbuscular mycorrhiza) AM symbiosis has been reported in tomatoes to confer reduced susceptibility to shoot pathogens such as *Alternaria solani* [25], *Xanthomonas campestris* pv. *vesicatoria* [14], and *Botrytis cinerea* [26], among others.

Several changes must occur locally and systemically in mycorrhizal plants in order to show resistance to pathogens. In this respect, this regulation has been shown to occur through different molecules that act in signaling pathways such as jasmonic acid (JA), salicylic acid (SA), abscisic acid (ABA), and ethylene (ET) [17,20,27]. Recently, Goddard et al. (2021) found strong induction of the expression of PR (pathogenesis-related) proteins in roots in grapevine inoculated with *Rhizophagus irregularis*, suggesting that these proteins could play a role in mycorrhiza development as well as conferring higher resistance to root pathogens. In leaves, metabolic changes induced by AM fungal colonization are less evident, and higher levels of linoleic and linolenic acids and lower levels of sucrose, quinic acid, and shikimic acid have been observed. Furthermore, mycorrhiza colonization is reported to result in enhanced JA and SA levels in foliar tissues [28]. In order to gain insight into the changes occurring in leaves of mycorrhizal plants, Cervantes-Gámez et al. (2016) performed a differential transcriptomic analysis of tomato leaves of colonized and non-colonized plants using the AMF *Rhizophagus irregularis* [15]. These authors found that several genes related to the cell wall are differentially regulated. In another study, mycorrhizal tomato plants also inoculated with *Rhizophagus irregularis* displayed callose accumulation following *Botrytis cinerea* infection. The fact that the callose inhibitor 2-deoxy-D-glucose abolished MIR confirms the relevance of callose to the bioprotection phenomena [26]. However, the mechanisms of mycorrhiza-induced systemic resistance responses, as well as defense priming, remain unknown.

MicroRNAs (miRNAs) are small non-coding RNA molecules of 21–24 nucleotides in length that regulate the expression of genes at the post-transcriptional level [29,30,31]. These regulatory molecules occupy a unique position within the hierarchy of genetic regulators; however, unlike conventional transcription factors, they can act as fine-tuning molecules of programmed transcription. The regulatory functions of miRNAs are essential for different biological processes in plants, such as development, metabolism, and the responses to biotic and abiotic stress [32,33,34,35,36,37,38,39]. The reason for this is that they can recognize specific regions of a target mRNA by base complementarity, promoting their cleavage by incorporating the RISC (RNA-induced silencing complex) with consequent silencing of the complementary messenger RNA, thereby inhibiting its subsequent translation [29,40]. The ability of miRNAs to repress their target genes depends on their expression levels [30]. However, the role of miRNAs in the priming induced by arbuscular mycorrhizal symbiosis in shoots has not yet been documented. We therefore analyzed miRNA expression in the leaves of mycorrhiza-colonized tomato plants in order to identify potential functions during these defense-enhancing responses in the aerial part of plants during this symbiosis.

In this work, we aimed to analyze the transcriptional and post-transcriptional responses related to the cell wall in order to identify players induced by mycorrhiza colonization potentially involved in defense priming. The results obtained here support the hypothesis that reinforcement of the cell wall plays a role in the priming mechanism triggered by mycorrhiza symbiosis, by strengthening the first physical barriers against pathogens.

## 2. Materials and Methods

### 2.1. Plant Growth and Tissue Collection

Tomato seeds (*Solanum lycopersicum* var. *Missouri*) were surface-sterilized for 5 min in 70% ethanol, and 30 min in 5% sodium hypochlorite. Next, seeds were rinsed five times in sterile distilled water. Seeds were planted in sterilized vermiculite/sand (3:1 *v*/*v*), and five days later, plantlets were inoculated with 400 spores of the AMF *R. irregularis* per plant (MYC). Control plants (CTR) were mock-inoculated with water from the last rinse of the spores, and grown under the same conditions as colonized plants. Plants were kept in a growth room (25 °C; 12 h light/12 h dark). Four weeks after planting, tomato plants were individually transplanted to 500-mL pots with the same substrate and maintained under the same growing conditions for four additional weeks. Tomato plants were therefore eight weeks old when harvested.

Plants were watered once per week with distilled water and twice per week with the following modified Hoagland’s solution: (Ca(NO_3_)_2_·4H_2_O, 2.5 mM; KNO_3_, 2.5 mM; MgSO_4_·7H_2_O, 1 mM; NaFe EDTA, 0.05 mM; H_3_BO_3_, 10 μM; Na_2_MoO_4_·2H_2_O, 0.2 μM; ZnSO_4_·7H_2_O, 1 μM; MnCl_2_·4H_2_O, 2.0 μM; CuSO_4_·5H_2_O, 0.5 μM; CoCl_2_·6H_2_O, 0.2 μM; HCL, 25 μM; MES buffer, 0.5 mM) [41]. The phosphate concentration of the solution was adjusted to 0.05 mM KH_2_PO_4_ to favor mycorrhiza colonization. Eight weeks after planting, plants were harvested. The shoots and roots of each plant were immediately frozen in liquid nitrogen and stored at −70 °C. All leaves were pooled from each plant and ground to a fine powder in liquid nitrogen. The experiment was repeated three times.

### 2.2. Sclerotinia Sclerotiorum Inoculum

Sclerotia collected in agricultural fields in the state of Sinaloa, Mexico, were surface sterilized for 1 min in 0.05% sodium hypochlorite and rinsed three times with sterile distilled water. Subsequently, sclerotia were placed in potato dextrose agar (PDA) plates and incubated at 19 °C for germination. Mycelia were transferred to fresh PDA plates and incubated for three more days. Mycelium agar discs (0.3 cm in diameter) from the active growing zone in the plate were used for infection experiments [14]. The molecular identification of *S. sclerotiorum* was performed by sequencing the ITS1, 5.8 S, and ITS2 regions of rDNA fragment amplicons, using the ITS1 and ITS4 primers (accession number ON430518).

### 2.3. Infection Assays of Tomato Leaves with the Foliar Pathogen S. sclerotiorum

Wet chambers were prepared by placing a wet paper towel on the bottom of Petri dishes. A tomato leaflet was placed in the Petri dish, and an agar disc containing *S. sclerotiorum* mycelium was placed on the leaflet. Petri dishes were sealed with parafilm and incubated at 19 °C. The level of infection was monitored by measuring the diameter of the necrotic lesions caused by the pathogen at 27 and 36 h post-infection (hpi). Since necrotic lesions were not perfect circles, we recorded the lengths of the longest and shortest axes, and the average of the two lengths was calculated and considered to be the diameter of the lesion. Two leaflets (from the second true leaf) per plant were used for the assay. Five mycorrhiza-colonized (MYC) and five mock-inoculated controls (CTR) were used in the experiments.

### 2.4. RNA Extraction

Total RNA from the leaves of three biological replicates (individual plants) of MYC and CTR plants was obtained using TRIzol^®^ reagent (Ambion; Carlsbad, CA, USA). The concentration of total RNA, as well as the A260/280 and A260/230 ratios, was estimated using a NanoDrop 2000 c Spectrometer (Thermo; Waltham, MA, USA). All RNA samples were treated with the Turbo DNA-free™ kit (Invitrogen by Thermo Fisher Scientific) to remove any genomic DNA contaminant before qRT-PCR analyses, according to the manufacturer’s instructions.

### 2.5. cDNA Synthesis and Quantitative RT-PCR (qPCR)

cDNA was synthesized from 300 ng of total RNA (Superscript III reverse transcriptase kit, Invitrogen, Waltham, MA, USA), using the manufacturer’s instructions. cDNA from miRNA was synthesized from 300 ng of total RNA with the MirX miRNA First-Strand Synthesis kit (Takara Bio, Kusatsu City, Japan), according to the manufacturer’s instructions.

qPCR reactions were performed in triplicate for each of the three biological replicates in a Rotor-Gene Q real-time PCR system (Qiagen, Venlo, The Netherlands). The total reaction volume per reaction was 10 μL, including 500 nM of each primer, 10 ng of cDNA and 5 μL of SYBR Green master mix (Qiagen). Non-template controls were included. The PCR program included an initial step at 95 °C (5 min), followed by 40 cycles of steps at 95 °C for 5 s and 60 °C for 10 s. Dissociation curves were performed at the end of each run.

The expression of ubiquitin and elongation factor 1α (*EF-1α*) was used for the normalization of gene expression, and the *U6* snRNA was used for miRNAs. All primers used in this work are listed in Table A1, Table A2 and Table A3. Primers were designed in the exon-exon junction regions. Relative gene expression was calculated according to the 2^−∆∆Ct^ method using the CTR treatment as the reference condition.

### 2.6. Determination of Mycorrhiza Colonization

**Figure A1:**
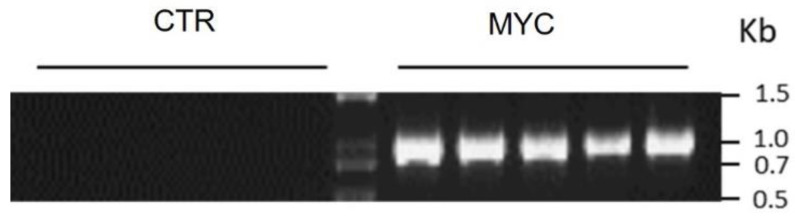
End-point PCR of cDNA obtained from mycorrhiza-colonized (MYC) and non-colonized (CTR) roots using the *SlPT* primers that hybridize to a mycorrhiza-specific phosphate transporter (Solyc06g051850) (forward: 5′-GAAGGGGAGCCATTTAATGTGG-3′; reverse: 5′-GATGGCCAGAGACAGGTTTG-3′).

To confirm mycorrhiza colonization, the root systems were collected and frozen immediately in liquid nitrogen. Total RNA was extracted and cDNA synthesized as explained above. End-point PCR was performed using these cDNA samples as templates and primers for the mycorrhiza-specific phosphate transporter gene (Solyc06g051850), according to Ho-Plágaro et al. (2018) [42]. No bands were detected in the roots of CTR plants, whereas a band corresponding to the expected PCR product size was observed in roots of MYC plants (a representative experiment is presented in Figure A1).

### 2.7. Cellulose Quantification

Ten leaflets per plant were pooled and lyophilized to determine cellulose content from MYC and CTR tomato plants. Cellulose determination was based on the method reported by Updegraff (1969) [43]. Fifty mg of lyophilized tissue was added to 1 mL of 80% ethanol and incubated at 80 °C for 1 h. Ethanol was then eliminated, and 200 µL of 90% DMSO was added and incubated for 1 h at room temperature, and finally centrifuged for 3 min at 2500 rpm. The pellet was rinsed twice in 96% acetone and three times in deionized water, and centrifuged again for 3 min at 2500 rpm. The residue was incubated with 5 U α-amylase (Sigma, Cat. No. A7095) in 100 mM ammonium formate for 72 h at room temperature. Acetone and water rinses were repeated. Then, the residue was added to 1 mL acetic acid:water:nitric acid (8:2:1 *v*/*v*/*v*) and heated at 100 °C for 30 min. The samples were allowed to cool down, and were then centrifuged at 2500 rpm for 5 min. The supernatant was decanted and the sediment was suspended in 1 mL of deionized water, centrifuged again, and the supernatant was discarded. Next, 1 mL of 72% sulfuric acid was added to the pellet and incubated at 50 °C for 1 h with agitation at 120 rpm, and then centrifuged at 2500 rpm for 5 min. The supernatant was transferred to a 10-mL volumetric flask and water was added to 10 mL. In a 1.5-mL Eppendorf tube, 10 µL of the flask content was mixed by inversion with 1 mL of cold anthrone solution (0.2 g of anthrone in 100 mL of 72% sulfuric acid) and left on ice for 2 min. The sample was then heated at 100 °C for 15 min and allowed to cool down. Spectrophotometric reads were taken on the samples at 620 nm, and a standard curve with cellulose was used.

## 3. Results

### 3.1. Leaves of AM Tomato Plants Are Less Susceptible to the Foliar Pathogen S. sclerotiorum

**Figure 1:**
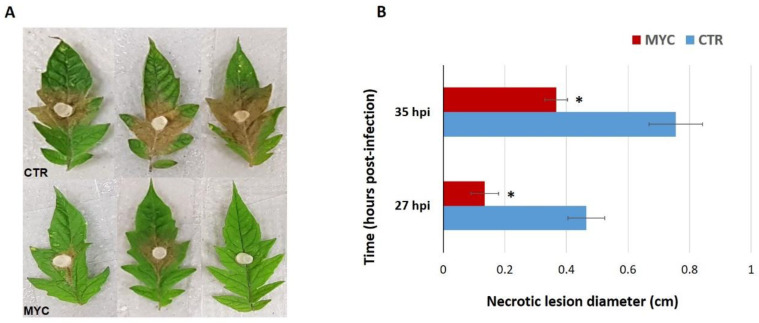
Mycorrhiza-induced resistance in tomato leaves. (**A**) *S. sclerotiorum* infection on tomato leaves estimated by measuring the diameter of the necrotic lesion on MYC and CTR plants at 35 hpi (hours post-infection). (**B**) Diameter of lesions caused by *S. sclerotiorum* on leaves of MYC and CTR tomato after 27 and 35 hpi. CTR, non-colonized plants; MYC, AM-plants colonized with *Rhizophagus irregularis*. (*) indicates significantly different means between CTR and MYC per time point (Student’s *t*-test, *p* ≤ 0.05, n = 5). Bars, ±SE.

**Figure A2:**
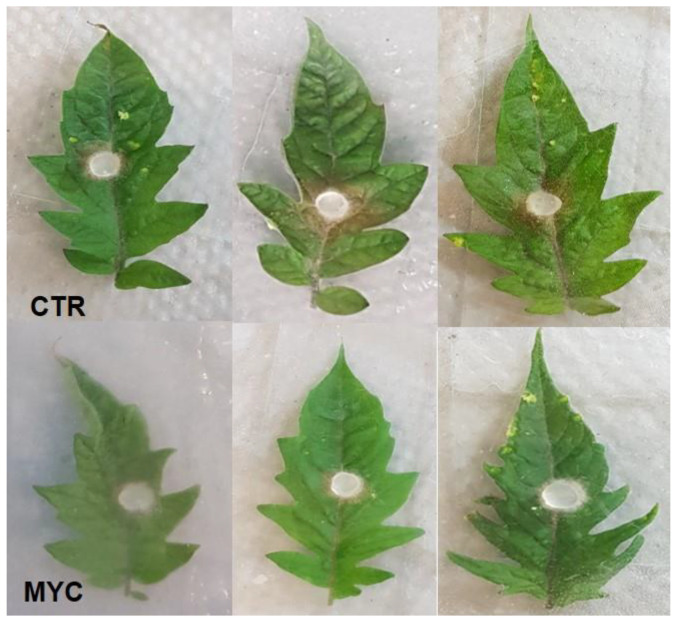
*S. sclerotiorum* infection on tomato leaves estimated by measuring the diameter of the necrotic lesion on MYC and CTR plants at 27 hpi (hours post-infection).

In order to confirm the reduction in susceptibility to a foliar pathogen by AM symbiosis, leaves of MYC and CTR plants were infected with *S. sclerotiorum* in detached leaf assays. The necrotic lesion diameters caused by *S. sclerotiorum* were significantly smaller in leaves of MYC plants as compared to CTR (Figure 1A). The infection became evident after 27 h of inoculation with the pathogenic fungus, showing a 70% smaller in diameter lesion size in MYC plants (Figure A2); at 35 h, the infection was 50% less than CTR plants (Figure 1B). These results confirm that AM tomato plants are less susceptible to the foliar pathogen *S. sclerotiorum* showing mycorrhiza-induced resistance, as previously reported in this plant species.

### 3.2. Expression of Genes Involved in the Cell Wall

**Figure 2:**
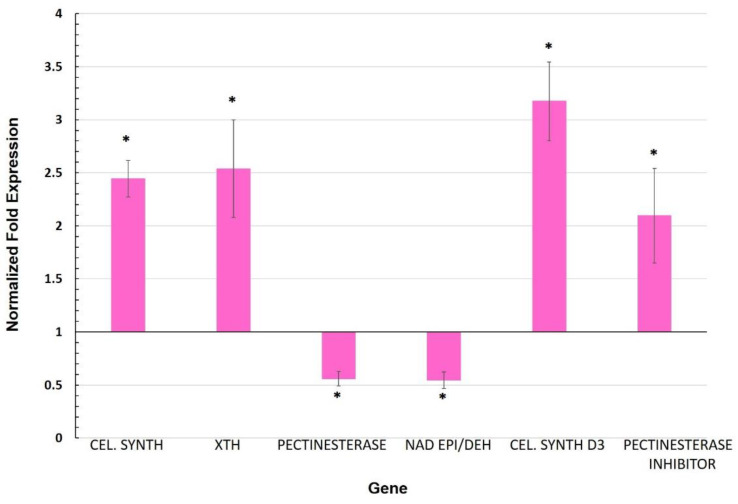
Normalized fold expression of cell wall-related genes in leaves of mycorrhizal tomato plants. Gene expression was determined by qRT-PCR (2^−∆∆Ct^). Values represent the average ±SE of the normalized relative expression (MYC/CTR) from three independent biological experiments (n = 3 per experiment). Ubiquitin was used as a reference gene for data normalization. (*) represent significantly different means between MYC/CTR according to the statistical analysis (Student’s *t*-test, *p* ≤ 0.05) Bars, ±SE. (CEL SYNTH: Cellulose synthase; XTH: Xyloglucan endotransglucosylase/hydrolase; NAD EPI/DEH: NAD dependent epimerase/dehydratase; CEL SYNTH D3: Cellulose synthase-like protein D3).

The focus of this research was to identify and characterize candidate genes involved in systemic priming by arbuscular mycorrhizal symbiosis in the leaves of tomato plants. Since one of the main functions of the cell wall is to act as a barrier against the attack of pathogens [44], this cellular structure could play a role in defense priming responses. Out of six cell wall-related genes that were reported as differentially expressed in a previous RNA-seq analysis [15], four of them were upregulated, including the gene that codes for cellulose synthase-like protein (Solyc03g097050). This gene displayed the highest induction (a 3.2-fold change as compared to CTR plants), followed by xyloglucan endotransglucosylase/hydrolase (Solyc09g008320; 2.5-fold change), cellulose synthase (Solyc07g051820; 2.4-fold change), and pectinesterase inhibitor (Solyc07g042390; 2.2-fold change) (Figure 2).

**Figure A3:**
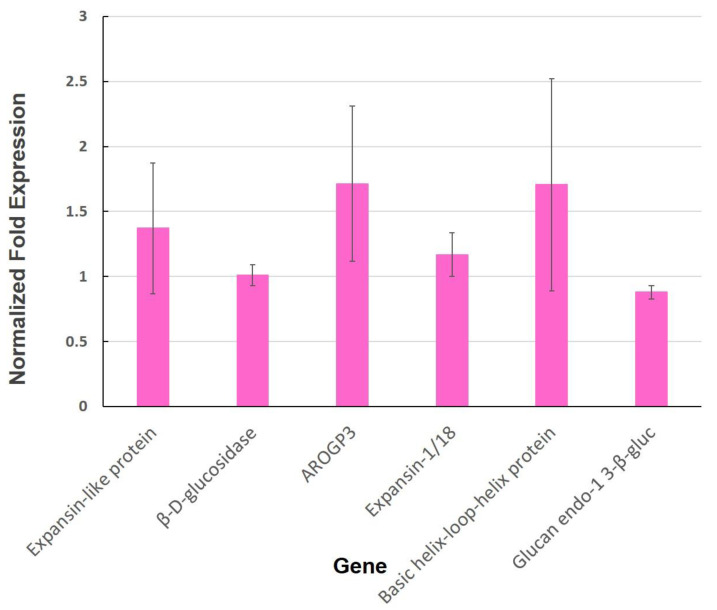
Normalized fold expression of cell wall-related genes in leaves of mycorrhizal tomato plants. Gene expression was determined by qRT-PCR (2^−∆∆Ct^). Values represent the average ±SE of the normalized relative expression (MYC/CTR) from three independent biological experiments (n = 3 per experiment). Ubiquitin was used as a reference gene for data normalization. No significantly different means were found between MYC/CTR according to the statistical analysis (Student’s *t*-test, *p* ≤ 0.05). Bars, ±SE.

For genes showing repression, we found that pectinesterase (Solyc06g009190) and NAD-dependent epimerase/dehydratase (Solyc12g010540) displayed decreased expression as compared to CTR (Figure 2). The genes expansin-like protein, β-D-glucosidase, AROGP3, expansin-1/18, basic helix-loop-helix protein, and glucan endo-1,3-β-gluc were also quantified, although no significant differential expression (*p* < 0.05) was detected (Figure A3).

### 3.3. Expression of Genes Involved in the Cuticle

**Figure 3:**
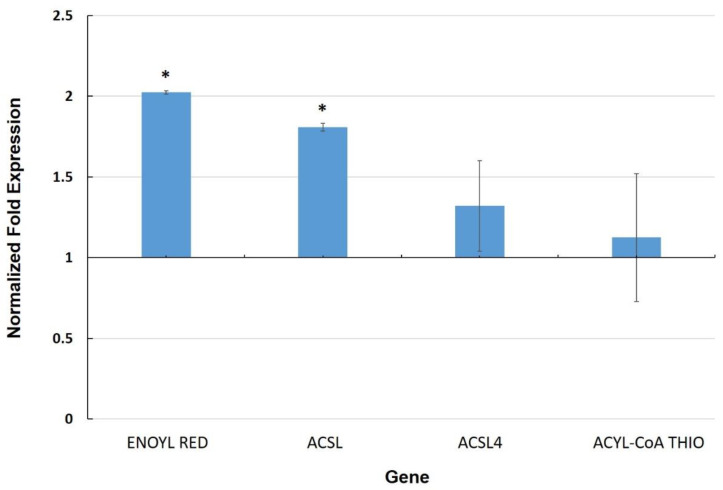
Normalized fold expression of cuticle-related genes in leaves of mycorrhizal tomato plants. Gene expression was determined by qRT-PCR (2^−∆∆Ct^). Values represent the average ±SE of the normalized relative expression (MYC/CTR) from three independent biological experiments (n = 3 per experiment). Ubiquitin was used as a reference gene for data normalization. (*) represent significantly different means between MYC/CTR according to the statistical analysis (Student’s *t*-test, *p* ≤ 0.05). Bars, ±SE. (ENOYL RED: Enoyl reductase; ACSL: Long-chain-fatty-acid-CoA ligase; ACSL4: Long-chain-fatty-acid-CoA ligase 4; ACYL CoA THIO: Acyl-CoA thioesterase).

The cuticle is a layer composed of lipid polymers and wax that coats the outer surface of epidermal cells, providing hydrophobicity to plant leaves and a physical barrier against pathogens as well as a related defense role [45]. We selected four genes based on a previous differential RNA-seq analysis between leaves of MYC and CTR plants [15]. These genes are related to fatty acid metabolism, which could be associated with the biosynthesis of cuticle components. Two of these genes, enoyl reductase (Solyc10g078740) and long-chain-fatty-acid-CoA ligase (Solyc03g025720), showed transcriptional upregulation in leaves of MYC plants as compared to CTR (Figure 3), whereas another long-chain-fatty-acid-CoA ligase (Solyc12g009040) and an acyl-CoA thioesterase showed no significant induction in MYC plants in our experiments.

### 3.4. Cellulose Content in Leaves of Mycorrhizal Tomato Plants

**Figure 4:**
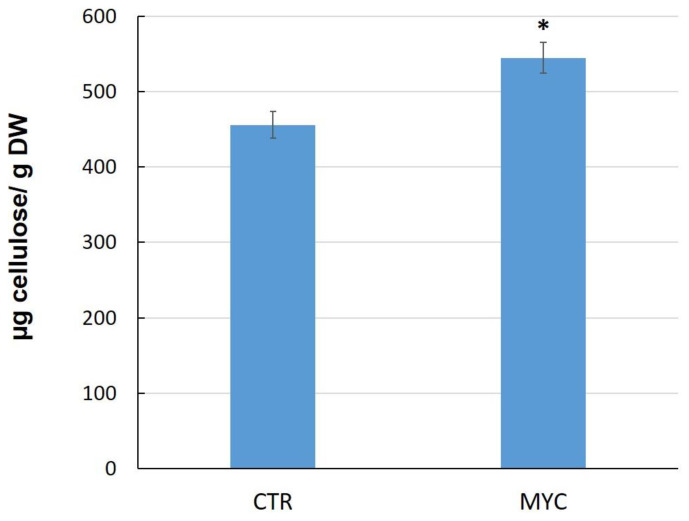
Cellulose content in leaves of MYC and CTR tomato plants. Values represent the average from two independent biological experiments (n = 5 per experiment). (*) represent significantly different means between MYC/CTR according to the statistical analysis (Student’s *t*-test, *p* ≤ 0.05). Bars, ±SE.

Since two cellulose synthases genes were upregulated in the leaves of MYC plants as compared to CTR plants, we next determined cellulose content in these tissues. The results show that leaves of MYC plants accumulate higher levels of cellulose than the controls by about 20% (Figure 4).

### 3.5. Expression Analysis of Selected miRNAs and Their Target Genes in Leaves of Mycorrhiza-Colonized Tomato

Considering that miRNAs are essential post-transcriptional regulators in response to biotic and abiotic stress, one of our goals was to identify differentially regulated miRNAs in leaves of MYC plants that could be relevant in defense priming in tomato, i.e., in MYC plants before any pathogen attack. Since there is no published information on miRNA in the leaves of mycorrhizal plants, we selected miRNAs that are reported to be differentially regulated in previous studies in mycorrhizal tomato roots [46], as well as miRNAs that were identified as targeting cell wall-related genes in other plants such as sorghum [47].

**Figure 5:**
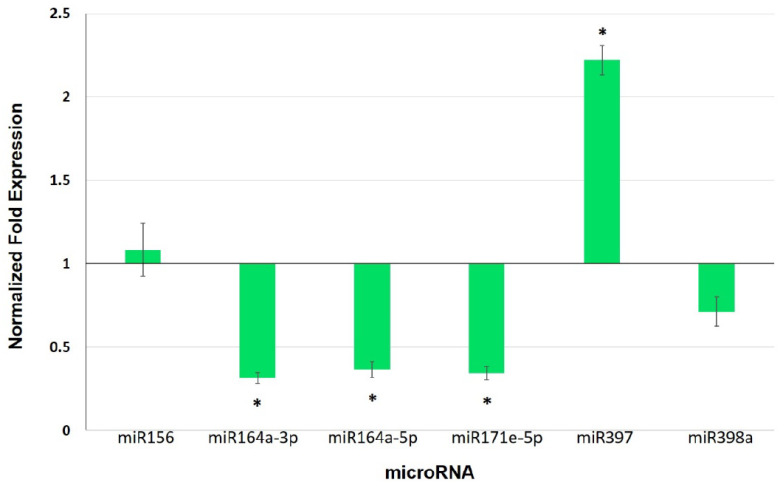
Normalized fold expression levels of selected miRNAs in leaves of mycorrhizal tomato plants. Expression was determined by qRT-PCR (2^−∆∆Ct^). Values represent the average ±SE of the normalized relative expression (MYC/CTR) from three independent biological experiments (n = 3 per experiment). U6 was used as a reference gene for data normalization. (*) represent significantly different means between MYC/CTR according to the statistical analysis (Student’s *t*-test, *p* ≤ 0.05). Bars, ±SE.

Using psRNATarget, we identified five miRNAs that have cell wall-related proteins as targets, and one, miR156, that targets cuticle (Table 1). We quantified the expression levels of these miRNAs, finding that miR64a-3p, miR164a-5p, and miR171e-5p were repressed (0.3-, 0.35- and 0.32-fold change, respectively), whereas miR397 was induced (2.2-fold change) (*p* < 0.05) (Figure 5).

**Figure 6:**
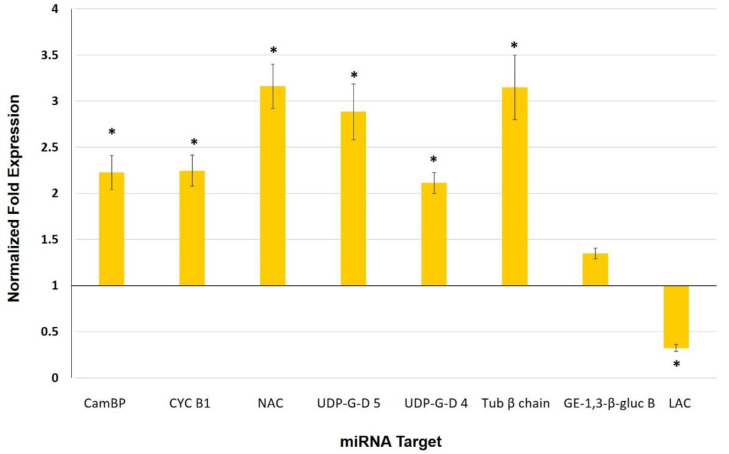
Normalized fold expression levels of miRNA target genes in leaves of mycorrhizal tomato plants. Expression was determined by qRT-PCR (2^−∆∆Ct^). Values represent the average ±SE of the normalized relative expression (MYC/CTR) from three independent biological experiments (n = 3 per experiment). Ubiquitin was used as a reference gene for data normalization. (*) represent significantly different means between MYC/CTR according to the statistical analysis (Student’s *t*-test, *p* ≤ 0.05). Bars, ±SE. (CamBP: Calmodulin binding protein; CYC B1: Cyclin B1; NAC: NAC domain protein; UDP-G-D5: UDP-glucoronic acid descarboxylase 5; UDP-G-D4: UDP-glucoronic acid descarboxylase 4; Tub β chain: Tubulin beta chain; GE-1,3-β-gluc B: Glucan endo-1,3-β-glucosidase B; LAC: Laccase).

To gain insight into specific roles of miRNAs in mycorrhizal tomato leaves, we measured the relative gene expression of the predicted genes targeted by qRT-PCR (Figure 6). None of the identified target proteins corresponded to any of the previously analyzed cell wall-related genes.

The identified targets for miR164a-3p were a calmodulin-binding protein and a cyclin B1; for miR164a-5p, a NAC domain protein, a UDP-glucuronic acid decarboxylase 5, and a UDP-glucuronic acid decarboxylase 4; for miR171e-5p, a tubulin β chain and a glucan endo-1,3-β-glucosidase B; and for miR397, a laccase (Table 1).

## 4. Discussion

### 4.1. Mycorrhiza Colonization Primes Tomato Plants for a Better Defense against a Foliar Pathogen

In our *S. sclerotiorum* infection tests on detached leaves of MYC tomato plants, the lesions were significantly smaller in diameter than the CTR plants (Figure 1). These results are in agreement with several reports, including Song et al. (2015), who reported that leaves of tomato colonized by *Funneliformis mosseae* displayed a significant reduction in the damage caused by *Alternaria solani* [48]. Furthermore, Mustafa et al. (2016) showed that *Blumeria graminis* infection in leaves of mycorrhiza-colonized wheat plants was reduced by 78% as compared to non-colonized plants [49]. Similarly, leaves of mycorrhizal rice plants showed a reduction in the severity of the disease caused by the pathogenic fungus *Magnaporthe oryzae* [27]. Fiorilli et al. (2018) observed that mycorrhiza-colonized wheat plants increased biomass and grain yield, and displayed a reduction in lesion area by *Xanthomonas translucens* infection [50]. In our own research group, this same pattern has been reported in both tomato and bean studies [14,15].

Although there is abundant evidence for the occurrence of mycorrhiza-induced resistance, much work must still be performed to elucidate the biochemical and molecular mechanisms involved when the mycorrhiza plant is defending itself against a pathogen, as well as before the attack occurs, i.e., during the priming stage. The discrete modification and/or remodeling of some cell wall components such as cellulose fibrils, lignin, and even cuticle could be important for mycorrhiza plants before their encounter with a pathogen, as a means to mount a faster and stronger defense reaction in a subsequent pathogen attack. In addition, it is well known that the cell wall is remodeled and reinforced at specific sites of interaction with pathogens [51].

### 4.2. Cell Wall and Cuticle Genes in Mycorrhiza Defense Priming

We observed that a cellulose synthase and a cellulose synthase-like protein were both upregulated in the leaves of MYC plants (Figure 2). Since this regulation was accompanied by an increase in cellulose content in leaves of MYC plants as compared to CTR (Figure 4), it is possible that that the proteins encoded by these genes might be involved in the reinforcement of the cell wall, as a priming response before any interaction with a pathogen. Cellulose, the main constituent of cell walls (40%), complexes with other components such as proteins, high molecular weight polysaccharides and aromatic substances, and is capable of dynamic changes [52,53,54].

Previous studies have linked the regulation of cellulose synthase genes to defense: in rice, cellulose synthase genes were markedly upregulated in an RSV (rice stripe virus)-resistant cultivar, but downregulated in a susceptible cultivar [55]. Cell wall strengthening has been widely documented in response to pathogen attack [56,57,58]. In addition, cellulose accumulation has been reported to occur in response to environmental stress, implying that the cell wall is reinforced to avoid external stress [59]. Here, however, we are reporting the accumulation of cellulose in leaf tissue that has not yet been attacked by a pathogen. We therefore take this accumulation to be a response to the establishment of symbiosis, which can be interpreted as a step in priming, since it occurs as a response to the interaction with a beneficial microorganism (in this case a mycorrhizal fungus) and before any attack by a pathogen.

Interestingly, we observed the downregulation of a pectinesterase gene in leaves of MYC plants, while a pectinesterase inhibitor gene was also upregulated (Figure 2). Pectinesterases catalyze the hydrolysis of pectin methyl esters, thus decreasing their degree of esterification, reducing intracellular adhesiveness and tissue rigidity. Pectinesterases have been shown to have an important function in response to fungal pathogens and are necessary for the systemic spread of tobacco mosaic virus throughout a plant [60,61]. In studies on cotton plants, pectinesterase inhibitor (PMEI) was shown to participate in plant responses to fungal infection, including *Verticillium dahliae* in cotton plants; its ectopic expression also increased pectin methyl esterification and limited fungal disease [62]. On the other hand, in pepper plants, silencing of the PMEI gene resulted in enhanced disease susceptibility to infection by the virulent *Xanthomonas campestris* pv. *vesicatoria*, whereas its overexpression in Arabidopsis showed enhanced resistance to *Pseudomonas syringae* pv. *tomato* [63]. These results suggest that the repression of a pectinesterase gene and the upregulation of a pectinesterase inhibitor may hinder the success of a subsequent pathogen infection, indicating that these two genes might be mycorrhiza priming genes.

Other genes that we found to be upregulated in leaves of MYC plants include an enoyl reductase and a long-chain-fatty-acid-CoA ligase (Figure 3), which are involved in fatty acid metabolism as well as the biosynthesis of cuticle components. In addition to its essential role in limiting water loss, the cuticle protects the plant against xenobiotics and pathogens. Components of the cuticle are perceived by pathogenic fungi, and they induce several processes during pathogenesis. Furthermore, modifications of the cuticle can result in resistance to necrotrophs [45]. Thus, alteration of the cuticle may also be part of the priming mechanism in leaves of mycorrhizal plants, as a means to more efficiently react to a subsequent pathogen attack.

### 4.3. miRNAs as Potential Players in Mycorrhiza Defense Priming

The function of miRNAs as post-transcriptional regulators suggests that they could play a role in the mycorrhizal systemic response in leaves. In the present work, three miRNAs (miR164a-3p, miR164a-5p, and miR171e-5p) were found to be repressed in leaves of MYC plants (Figure 5), whereas their predicted target genes (Table 1) exhibited an inverse expression relative to their corresponding miRNAs (Figure 6), suggesting that these miRNAs could have a post-transcriptional regulatory action on the predicted targets. This miRNA/target gene expression pattern indicates that these molecular entities might be part of the response of the shoot to AM establishment in the root that is involved in defense priming.

One of the predicted target genes of miR164a-3p is a calmodulin-binding protein (CaMBP). These genes belong to the IQD/SUN gene family, and in *Populus* plants an IDQ/SUN gene has been associated with the signaling of cell wall biosynthesis [64]. Plant resistance to diseases involves a number of signaling response pathways including calcium/calmodulin, reactive oxygen species, and phytohormones [65]. Calmodulin regulates plant disease responses through CaMBPs, often by affecting the biosynthesis or signaling of JA and SA [66]. The induction of CaMBPs has been associated with defense responses in rice against *M. grisea* [67], and in Arabidopsis against *Botrytis cinerea* through the regulation of JA synthesis [66]. On the other hand, jasmonates are reported to accumulate in leaves on mycorrhiza-colonized plants [11,16,28], which could be mediated by a CaMBP such as the one induced in the present work. Although the genome-wide identification and expression analysis of this gene family in tomato has been published [68], no information on the role of these genes or their associated miRNAs in AM colonized plants has been reported. However, its potential role in the signaling of cell wall biosynthesis could be in accordance with its involvement in cell wall reinforcement by mycorrhizal priming.

Cyclin B1, another predicted target of miR164a-3p, also showed an inverse expression (upregulation) to this miRNA (Figure 6). The expression of cyclin B1 in Arabidopsis accelerates the proliferation of root cells and promotes DNA repair [69,70]. Recent studies by Ambastha and Leshem (2020) provide evidence for the intense activity of cyclin B1 during salt stress as a means to repair damaged DNA in Arabidopsis [71]. Since mycorrhiza colonization also induces resistance to abiotic stress, cyclin B1 might be involved in the response to abiotic instead of biotic stress.

Another downregulated miRNA in the leaves of MYC plants is miRNA164a-5p, and we detected the upregulation of one of its predicted targets, a NAC domain protein (Figure 6). NAC is an important family of plant transcription factors that are associated with developmental processes, and together with miR164 they are required for the subsequent formation of the limits of the lateral organs in the apical meristem, in addition to leaf development in tomato plants [72]. NACs are also implicated in leaf senescence and secondary wall formation, as well as responses to abiotic and biotic stresses [73,74,75]. In rice, the NAC TF gene (ONAC063) has been shown to respond to high-temperature stress (Yokotani et al. 2009) [76]. Similarly, overexpressing the NAC transcription factor JUNGBRUNNEN1 (JUB1; ANAC042) extends longevity and increases tolerance to heat stress in *Arabidopsis thaliana* [77]. Furthermore, the induction of miR164 and repression of its respective target gene in response to aluminum toxicity have been observed in nodulated bean plants [37].

The expression and regulation of lignin biosynthetic genes are determined by various transcription factors including NACs, whose repression leads to a reduction in secondary wall thickening in fibers [78,79,80]. The secondary wall is mainly composed of lignin, a polyphenolic biopolymer responsible for contributing to cell rigidity and protection against pathogens, as well as covering the interior of the vessels to facilitate hydrophilic transport.

NAC transcription factors also play an important role in the regulation of plant defense responses during pathogen stress, such as insect wounds [75,81,82] and nematode interaction [83]. In addition to the participation of NAC proteins in the defense mechanism against pathogens, the possible regulation of lignin biosynthesis by miR164a-5p during mycorrhiza colonization could be part of the priming mechanism. This in turn could result in the reinforcement of the cell wall to provide better defense in a subsequent pathogen attack, as shown in the present work.

UDP-glucuronic acid decarboxylases 4 and 5 were induced in leaves of MYC plants (Figure 6) whereas miR164a-5p was repressed (Figure 5), suggesting that UDP-glucuronic acid decarboxylases 4 and 5 genes are the target. The enzyme UDP-glucuronic acid decarboxylase is responsible for converting UDP-glucuronic acid (UDP-GLcA) into UDP-xylose, and is also involved in the formation of xylans during the biosynthesis of non-cellulosic polysaccharides of the cell wall [84,85]. Xylans play an important role in the integrity of the plant cell wall and increase its resistance to enzymatic digestion, thus helping plants to defend themselves against herbivores and pathogens. Crowe et al. (2021) demonstrated that xylans are critical for the proper bundling and alignment of cellulose microfibrils in plant secondary cell walls [86].

The interactions between the three main structural biopolymers xylan, cellulose and lignin seem to be essential for providing the rigidity of plant cell walls [87,88]. The possible regulation of two UDP-glucuronic decarboxylases and a NAC domain protein by miR164a-5p, along with the accumulation of cellulose possibly due to the upregulation of cellulose synthase genes, could therefore be part of the priming responses that favor secondary cell wall reinforcement, even occurring before the pathogen attack.

MiR171e-5p was also identified as being repressed in the leaves of MYC plants (Figure 5), and one of its targets showing upregulation was a tubulin β chain gene (Figure 6). Tubulin is an essential protein for eukaryotic cells formed by two homologous globular proteins, α-tubulin and β-tubulin. This protein plays a major role in defining the shape of the cell and organizing its cytoplasm, as well as in cell division and the orientation of components in the cell wall [89,90]. Spokevicius et al. (2007) showed that β-tubulin is involved in determining the orientation of cellulose microfibrils in plant secondary fiber cell walls; in this way, β-tubulins participate in the flexibility, strength, and resistance of plants [91]. Together with the aforementioned analyses, our findings indicate that miR171 and its regulation of β-tubulin could participate in priming by mycorrhiza colonization to result in an improved defense after a pathogen attack, due to the structural role of β-tubulin in the orientation of cellulose microfibrils, an essential part of the secondary cell wall.

Another selected miRNA, miR397, showed upregulation expression (Figure 5), whereas its target, a laccase gene, was downregulated (Figure 6). Laccases are a family of multicopper oxidoreductase enzymes reported to be involved in the biosynthesis of lignin by polymerizing monolignols into lignin [92]. Consequently, their downregulation should imply a reduction in lignin polymerization. Interestingly, Lee et al. (2012) reported that lignin content was lower in arbuscular mycorrhizal plants as compared to non-mycorrhizal controls in drought-stressed perennial ryegrass [93]. Furthermore, Baslam et al. (2013) observed that the leaves of mycorrhizal alfalfa plants exposed to CO_2_ showed increased hemicellulose and decreased lignin concentrations in cell walls as compared to non-mycorrhizal plants [94]. Determining the lignin content in leaves of mycorrhizal tomato will help support the hypothesis that this laccase, regulated by miR397, has an effect on lignin and its potential role in priming. Although the downregulation of laccase and upregulation of NAC domain protein seem to have contrasting effects on lignin content, these two potential strategies for regulating lignin composition might reflect that more than one mechanism is needed to fine-tune the accumulation of such an important cell wall component as lignin.

In contrast, Pan et al. (2017) observed in rice that miR397 negatively regulates laccases, which induced the expression of three oxidase/peroxidase genes and promoted an increase in herbicide tolerance related to a better response to oxidative stress. This places miR397 as a potentially important element in the regulation of responses to oxidative stress [95], which could certainly have a role in the plant response to mycorrhiza colonization, as previously noted [96].

It is important to mention that some of the analyzed targets have already been validated by degradome sequencing in other tomato studies, such as the case of NAC domain protein as a target of miR164a-5p [97], and laccase as a target of miR397 [98].

Although there is abundant evidence for the role of cell wall modifications as an important mechanism in plant defense against pathogens, our results support the idea that the priming mechanism induced by arbuscular mycorrhiza colonization also involves the reinforcement of the cell wall. Additional studies will be needed in order to completely elucidate the role of the cell wall in the priming mechanism induced by mycorrhiza colonization.

## 5. Conclusions

The present work demonstrates that mycorrhiza colonization induces a priming state that confers an increase in defense against the foliar pathogen *Sclerotinia sclerotiorum* in tomato leaves. This could be associated with the observed differential expression of cell wall- and cuticle-related genes, as these two structures form the first physical barriers to pathogens. The induction of cellulose synthase genes is consistent with the higher cellulose content observed in the leaves of mycorrhizal plants as compared to control plants. Modifications of the cell wall and cuticle in response to the establishment of the symbiosis could be part of the priming mechanisms that prepare the plant tissues to respond in a faster and stronger manner to a subsequent pathogen encounter. Although abundant scientific work supports the idea that the cell wall is strengthened during a pathogen attack, literature concerning cell wall reinforcement in leaves as a result of mycorrhiza symbiosis is scarce.

In this study, we identified differentially expressed miRNAs/targets for the first time in the leaves of mycorrhizal tomato plants. These molecules could be part of a sophisticated and efficient strategy to improve defense induced by mycorrhiza colonization. Further analyses can build upon this work to demonstrate the specific roles of candidate tomato genes and miRNAs/target nodes in the priming mechanism, using genetic and/or functional approaches.

## Figures and Tables

**Table 1:** Target gene prediction of selected tomato miRNAs.

miRNA	Target	Gene	Expectation
sly-miR156	Acyl-CoA dehydrogenase family	Solyc05g054370.2.1	1.5
sly-miR164a-3p	Calmodulin-binding protein	Solyc09g082560.2.1	2.0
	Cyclin B1	Solyc01g009040.2.1	3.0
sly-miR164a-5p	NAC domain protein	Solyc07g066330.2.1	1.0
	UDP-glucuronic acid decarboxylase 5	Solyc11g066150.1.1	1.5
	UDP-glucuronic acid decarboxylase 4	Solyc01g066710.2.1	3.0
sly-miR171e-5p	Tubulin β chain	Solyc03g025730.2.1	2.5
	Glucan endo-1,3-β-glucosidase B	Solyc01g059980.2.1	3.0
sly-miR397	Laccase	Solyc07g049460.2.1	0.5
sly-miR398a	Endoglucanase 1	Solyc08g083210.2.1	3.0
	Endoglucanase 1	Solyc09g075360.2.1	3.5

Bioinformatic information was analyzed using psRNATarget.

## Data Availability

The data presented in this study are available on request from the corresponding author.

## Appendix A

**Table A1:** Primers Used for qPCR of Cell Wall- and Cuticle-Related Genes.

Gene	Accession ID	Fwd	Rv
Cellulose synthase	Solyc07g051820	TGGGGTCAAAAGTTAGGCTGG	CGGGTCGGGTAAACAGTAGG
Expansin-like protein	Solyc08g077330	AACTCTCAAACATGCCCGGA	TCCACAACTCCCAGTTTCTGT
β-D-glucosidase	Solyc11g071640	TCGGTAGTCAGGAGCATAGAGA	GAGCAGTGGGTGACTAGGTG
AROGP3	Solyc05g005540	CTTCCTTCCTGCATCTTACTTCT	CATCTTTAGCCACAACAACATCC
Expansin-1/18	Solyc06g076220	CCCTGGTGTTTTTACTGCCG	GCTCCACCCATAGTACCAGA
Xyloglucan endotransglucosydase/hydrolase	Solyc09g008320	TGGGGTCCTAATCACCAGAGT	CGACTTGAATCCACTGCCTGA
Pectinesterase	Solyc06g009190	CAAGGCGGGAACGTATTTCG	CATTTCCCACCACAGCAACG
NAD-dependent epimerase	Solyc12g010540	CGGTGTTTCGCTTCAACGAG	TGTGGAGGAGAGATACCGGG
Basic helix-loop-helix	Solyc09g083360	AACTAAGAGTGGAGCAGCAGA	TCAATGGGCACGAAGGTTCC
Cellulose synthase-like protein D3	Solyc03g097050	TGCTGGAATAATACAGGTGATGT	AACAAGCATGGGAAGACGGA
Glucan endo-1,3-β glucosidase	Solyc07g047710	AAATTTCAGTGAATGGGCAGC	TTGCTACAATCTGCACCCCC
Pectinesterase inhibitor	Solyc07g042390	TCGAGCAGGTAAAGCGTCTG	TCCTCCATCGAGTCACCCAT
Enoyl reductase	Solyc10g078740	AGTGACACAAAAGTCCTGGCA	CTTTTGCTGCACGACTTCCC
Long-chain-fatty-acid-CoA ligase	Solyc03g025720	AGCATTTGGGGCTCCTGTTT	CGACCCGGGAATATGAGGTC
Long-chain-fatty-acid-CoA ligase 4	Solyc12g009040	TGGGAGAGTTACGGGCAAAC	GCGGAAGCATCCCTGAAATG
Acyl-CoA thioesterase	Solyc01g094550	AGGGAGAGCTCGTAGCGT	ACTGCTAATGAACCTGTAGTGAC

## Appendix B

**Table A2:** Primers Used for miRNA qPCR.

miRNA	Primer
sly-miR156d-3p	GCTCACTGCTCTATCTGTCACC
sly-miR164a-3p	CATGTGCCTGTTTTCCCCATC
sly-miR164a-5p	TGGAGAAGCAGGGCACGTGCA
sly-miR171e-5p	AGATATTGATGCGGTTCAATC
sly-miR397-5p	ATTGAGTGCAGCGTTGATGA
sly-miR398a	TATGTTCTCAGGTCGCCCCTG

## Appendix C

**Table A3:** Primers Used for qPCR of Predicted miRNA Targets.

Gene	Accession ID	Fwd	Rv
Calmodulin-binding protein	Solyc09g082560.2.1	GCGATCCAATTCACTGCTGC	TCAGGGCTTTTCTTGCCAAAT
Cyclin B1	Solyc01g009040.2.1	GGCATCAGACAATCTTGCACC	AACTCCACAAGCAGCCTTGC
UDP-glucuronic acid decarboxylase 4	Solyc01g066710.2.1	CAGTGCTTCTGTGTCCGTTG	GGCACCTTTCCACCTGCATTA
UDP-glucuronic acid decarboxylase 5	Solyc11g066150.1.1	GTGACAGAGCCCTTGTTGGT	GCAGAATCCTTGCTCCGACA
NAC domain containing protein	Solyc07g066330.2.1	GTGGAATCCAAATTACCACCAGG	CAACACATGCCACTTCAGGA
Glucan endo-1,3 β-glucosidase B	Solyc01g059980.2.1	CAACATTCACATAACAGAGGCTCA	ATGTGATGGCAAGTTGTTCCC
Tubulin β-1 chain	Solyc03g025730.2.1	CGGAACTTATCGACTCGGTTATG	CCTGAAAACCTTGTAAGCAATCA
Laccase	Solyc07g049460.2.1	CCCTTGCTCCGTTAATCAAACA	TCCGTGACGTAGGGATCAGT
